# Investigation of Preanalytical Variables Impacting Pathogen Cell-Free DNA in Blood and Urine

**DOI:** 10.1128/JCM.00782-19

**Published:** 2019-10-23

**Authors:** Kanagavel Murugesan, Catherine A. Hogan, Zaida Palmer, Byron Reeve, Grant Theron, Alfred Andama, Akos Somoskovi, Amy Steadman, Damian Madan, Jason Andrews, Julio Croda, Malaya K. Sahoo, Adithya Cattamanchi, Benjamin A. Pinsky, Niaz Banaei

**Affiliations:** a Department of Pathology, Stanford University School of Medicine, Stanford, California, USA; b Clinical Microbiology Laboratory, Stanford Health Care, Stanford, California, USA; c NRF/DST Centre of Excellence for Biomedical Tuberculosis Research, South African Medical Research Council Centre for Tuberculosis Research, Division of Molecular Biology and Human Genetics, Faculty of Medicine and Health Sciences, Stellenbosch University, Cape Town, South Africa; d College of Health Sciences, Makerere University, Kampala, Uganda; e Global Health Technologies, Global Good Fund, Intellectual Ventures Laboratory, Bellevue, Washington, USA; f Intellectual Ventures Laboratory, Bellevue, Washington, USA; g Division of Infectious Diseases and Geographic Medicine, Department of Medicine, Stanford University School of Medicine, Stanford, California, USA; h Faculty of Health Sciences, Federal University of Grande Dourados, Dourados, Brazil; i Oswaldo Cruz Foundation, Campo Grande, Brazil; j Department of Medicine, University of California, San Francisco, San Francisco, California, USA; Johns Hopkins University School of Medicine

**Keywords:** cell-free DNA, liquid biopsy, PCR, preanalytical

## Abstract

Pathogen cell-free DNA (pcfDNA) in blood and urine is an attractive biomarker; however, the impact of preanalytical factors is not well understood. Blood and urine samples from healthy donors spiked with cfDNA from Mycobacterium tuberculosis, Salmonella enterica, Aspergillus fumigatus, and Epstein-Barr virus (EBV) and samples from tuberculosis patients were used to evaluate the impact of blood collection tube, urine preservative, processing delay, processing method, freezing and thawing, and sample volume on pcfDNA. The PCR cycle threshold (*C_T_*) was used to measure amplifiable cfDNA. In spiked samples, the median *C_T_* values for M. tuberculosis, S. enterica, and EBV cfDNA were significantly lower in blood collected in K_2_EDTA tubes than those in Streck and PAXgene blood collection tubes, and they were was significantly lower in urine preserved with EDTA (EDTA-urine) than in urine preserved with Streck reagent (Streck-urine). Blood and urine samples from TB patients preserved with K_2_EDTA and Tris-EDTA, respectively, showed significantly lower median M. tuberculosis *C_T_* values than with the Streck blood collection tube and Streck urine preservative. Processing delay increased the median pathogen *C_T_* values for Streck and PAXgene but not K_2_EDTA blood samples and for urine preserved with Streck reagent but not EDTA. Double-spin compared with single-spin plasma separation increased the median pathogen *C_T_* regardless of blood collection tube. No differences were observed between whole urine and supernatant and between fresh and thawed plasma and urine after 24 weeks at –80°C. Larger plasma and urine volumes in contrived and patient samples showed a significantly lower median M. tuberculosis *C_T_*. These findings suggest that large-volume single-spin K_2_EDTA-plasma and EDTA-whole urine with up to a 24-h processing delay may optimize pcfDNA detection.

## Abstract (precis)

Pathogen cell-free DNA (pcfDNA) in blood and urine is an attractive biomarker; however, the impact of preanalytical factors is not well understood. Blood and urine samples from healthy donors spiked with cfDNA from Mycobacterium tuberculosis, Salmonella enterica, Aspergillus fumigatus, and Epstein-Barr virus (EBV) and samples from tuberculosis patients were used to evaluate the impact of blood collection tube, urine preservative, processing delay, processing method, freezing and thawing, and sample volume on pcfDNA.

## INTRODUCTION

Analysis of cell-free DNA (cfDNA) in the acellular fraction of plasma and urine, also known as “liquid biopsy,” has emerged in the past decade as a promising new modality for noninvasive testing for conditions such as prenatal genetic abnormalities (1) and cancer driver mutations (2, 3). In the field of infectious diseases, the detection of Epstein-Barr virus (EBV) cfDNA in plasma has been in clinical use for several decades as a screening and prognostic test for EBV-associated nasopharyngeal carcinoma (4, 5). EBV cfDNA in plasma has been shown to be superior to cellular EBV as a marker of EBV-related diseases, particularly, posttransplant lymphoproliferative disorder (6). Pathogen cfDNA has also been applied to diagnose invasive infectious diseases. A number of studies have reported on the performance of targeted cfDNA assays for the diagnosis of tuberculosis (TB) (7–12), invasive fungal infections (13–16), and invasive parasitic infections (17). More recently, metagenomic next-generation sequencing of plasma cfDNA was evaluated in patients with bloodstream infection, cardiac surgery-associated Mycobacterium chimaera infection, and invasive fungal infection (18–22). Despite the growing interest, it is unclear how the biology and immunopathogenesis of each pathogen impact the availability of its cfDNA as a diagnostic biomarker. Furthermore, although noninvasive diagnosis of infectious diseases using cfDNA is an attractive premise, particularly in resource-poor settings, further research is needed to optimize preanalytical and analytical variables to define best practices and maximize assay performance (23).

In the field of oncology and obstetrics, much progress has been made in investigating and optimizing preanalytical factors that negatively impact the analysis of tumor and fetal cfDNA, respectively. Rapid processing of blood within 6 h of collection and using the standard K_2_EDTA blood collection tube were shown to be essential for preventing the dilution of tumor cfDNA with genomic DNA (gDNA) due to postcollection lysis of white blood cells (WBCs) (24–26). Fetal cfDNA was shown to remain stable up to 24 h at room temperature after blood collection in a K_2_EDTA blood collection tube (27). The storage of blood in K_2_EDTA tubes at 4°C was insufficient to prevent the dilution of tumor cfDNA (25). To mitigate tumor and fetal cfDNA dilution, cfDNA blood collection tubes, such as Cell-Free DNA BCT (Streck, Omaha, NE), the PAXgene blood circulating cell-free DNA (ccfDNA) tube (Qiagen, Germantown, MD), and a CellSave preservative tube (CellSearch, Huntington Valley, PA), have been developed and commercialized to stabilize WBCs *ex vivo* and enable delayed blood processing at room temperature up to 7 days without compromising tumor cfDNA fraction (24–26, 28). Similarly, urine preservatives, such as the Streck Cell-Free DNA urine preserve, has been developed to preserve cfDNA. The separation of plasma from the cellular fraction using double-spin versus single-spin methods has been shown to reduce the dilution of tumor cfDNA (26). Studies have also positively correlated the yield of tumor cfDNA to plasma volume used for extraction (26). Whether these reagents and processing methods uniformly apply to pathogen cfDNA is unclear. Unlike tumor cfDNA assays, which are designed to detect mutant allele in an abundant background of wild-type allele, targeted pathogen nucleic acid amplification tests (NAATs) are designed to amplify a highly specific pathogen sequence with no competition from a “wild type allele.” Thus, rapid processing, double-spin plasma separation, and stabilization of WBCs using expensive reagents and complex methods may not be critical for pathogen cfDNA. However, other preanalytical factors, such as sample volume, may be equally vital to the sensitive detection of pathogen cfDNA.

The aim of this study was to use contrived and clinical samples to investigate the impact of preanalytical variables, such as type of blood collection tube or urine preservative, processing delay, processing method, freezing and thawing, and sample volume on pathogen cfDNA detection in plasma and urine.

## MATERIALS AND METHODS

### Ethics.

This study was approved by the institutional review board at Stanford University. Approval for the collection of clinical samples was obtained from the institutional review board at the Federal University of Grande Dourados (UFGD) and the Comissão Nacional de Ética em Pesquisa in Brazil, the Stellenbosch University Faculty of Health Sciences Research Ethics Committee (N14/10/136) in South Africa, the Committee on Human Research at the University of California, San Francisco (UCSF), the Research Ethics Committee at the Makerere University School of Medicine Research, and the Uganda National Council for Science and Technology. All participants were >18 years of age and provided written informed consent.

### Study design.

**FIG 1:**
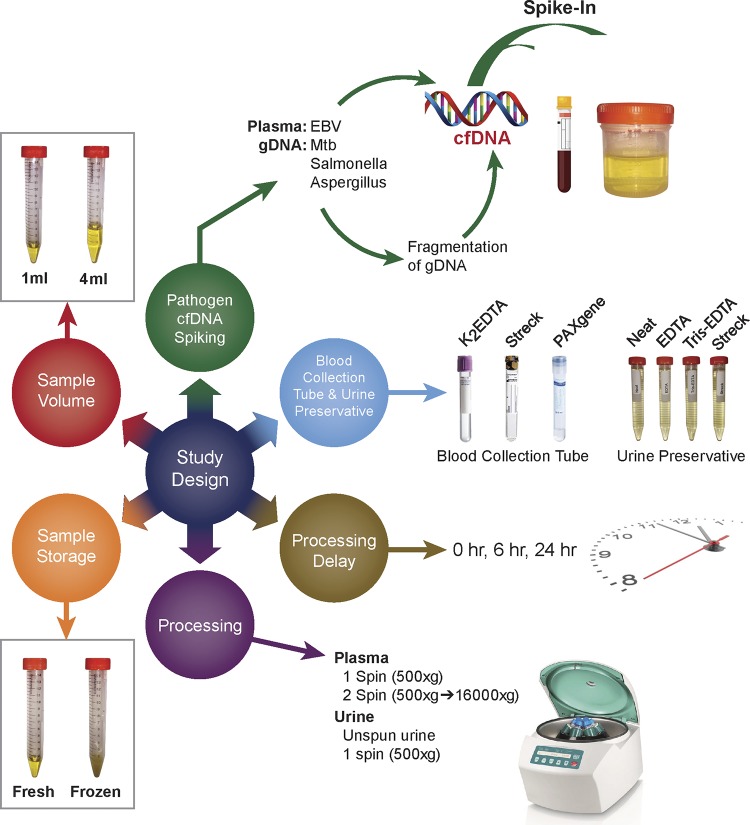
Study design. Spiking experiments with pathogen cfDNA from M. tuberculosis (Mtb), S. enterica (*Salmonella*), A. fumigatus (*Aspergillus*), and Epstein-Barr virus (EBV) were performed with fresh blood and urine from healthy donors to evaluate the impact of blood collection tube and urine preservative (blue circle), processing delay (brown circle), processing method (purple circle), sample storage (orange circle), and sample volume (red circle) on pathogen cfDNA in plasma and urine. In addition to using contrived samples, blood collection tube and urine preservative (blue circle) and sample volume (red circle) were evaluated using blood and urine samples from TB patients.

Spiking experiments with pathogen cfDNA from Mycobacterium tuberculosis, Salmonella enterica, Aspergillus fumigatus, and Epstein-Barr virus (EBV) were performed with fresh blood and urine from healthy donors to evaluate the impact of (i) blood collection tube and urine preservative, (ii) processing delay, (iii) processing method, (iv) freezing and thawing, and (v) sample volume on pathogen cfDNA in plasma and urine. Blood and urine from pretreatment TB patients collected under a different protocol at each site were used to validate findings from spiking experiments. A schematic overview of the study design is shown in Fig. 1.

### Study participants.

Ten healthy health care workers (5 females and 5 males between 23 and 46 years old of White, Asian, and Middle Eastern race) with no symptoms or signs of infection were recruited from the clinical laboratories at Stanford Health Care for blood and urine collection. Patients with confirmed pulmonary tuberculosis (TB) based on culture and/or the Xpert MTB/RIF assay (Cepheid, Sunnyvale, CA, USA) were recruited from UFGD hospital and mass screening studies in the Dourados prison in Brazil for blood collection, the Kiruddu Hospital and Kisenyi Healthcare Center level IV in Uganda for urine collection, and the Scottsdene and Wallacedene Community Health Centres in South Africa for blood collection.

### Pathogen cfDNA preparation.

Based on the fact that eukaryotic plasma cfDNA is ≈168 bp and that bacterial cfDNA in a septic patient was shown to be slightly shorter than 168 bp, we aimed to generate 200-bp cfDNA fragments for the spiking studies (29, 30). Genomic DNA from M. tuberculosis and S. enterica was extracted with a QIAamp DNA minikit (Qiagen), and A. fumigatus DNA was extracted with PrepMan Ultra sample preparation reagent (Applied Biosystems, Foster City, CA). DNA was digested with Fragmentase (New England BioLabs, Ipswich, MA), according to the manufacturer’s instructions. DNA fragments were separated on a 1.2% agarose gel using electrophoresis, and the region corresponding to 200 bp was cut and gel purified using the Wizard SV gel and PCR clean-up system (Promega, Madison, WI). A plasma sample from an infected patient containing naturally occurring EBV cfDNA at 2,000 IU/ml was used directly. DNA extracts and the plasma sample were serially diluted 1:10 in water, 3 μl of each dilution was spiked into 1 ml of donor plasma and urine, and the entire volume was extracted with the Maxwell RSC ccfDNA plasma kit (Promega) on the Maxwell RSC system. Real-time PCR was performed using the primers and probes shown in Table S1 in the supplemental material. PCRs consisted of 0.5 μM each primer and 0.2 μM each probe, 5 μl of 2× FastStart TaqMan Probe mastermix (Roche Applied Science, Indianapolis, IN), and 3 μl of DNA extract. The total volume was 10 μl per reaction. The reactions were run on a magnetic induction cycler (Bioline, Taunton, MA), with the following cycling parameters: 95°C for 10 min and 45 cycles of 95°C for 15 s, 60°C for 30 s, and 72°C for 30 s. Detection was performed in the green, yellow, orange, and red channels at 72°C. The threshold was set at 0.2 for all channels. The dilution that produced a cycle threshold (*C_T_*) between 25 and 30 was chosen for spiking experiments. Aliquots were stored at –20°C and thawed only one time for spiking studies.

### cfDNA spiking.

Within 10 min of blood and urine collection, samples were spiked with pathogen cfDNA at 3 μl per ml of blood and urine and gently mixed.

### Blood collection tube and urine preservative.

For spiking experiments, venipuncture blood was collected in three sets of K_2_EDTA (Becton, Dickinson, Franklin Lakes, NJ), Streck Cell-Free DNA BCT (Omaha, NE), and PAXgene blood ccfDNA (PreAnalytiX GmbH, Hombrechtikon, Switzerland) blood collection tubes. TB patients in South Africa were drawn concurrently in K_2_EDTA and Streck tubes and processed immediately. Plasma was frozen at –80°C and shipped to Stanford University for testing.

For an investigation of urine preservative in spiking experiments, urine collected in a collection cup was immediately transferred to four sets of conical tubes for each preservative and raw urine specimen. Urine specimens were treated with preservative to obtain a final concentration of 25 mM EDTA using 0.5 M EDTA (pH 7.6) stock (Sigma-Aldrich), 10 mM Tris-EDTA using 0.5 M Tris-HCl (pH 8.5), and 0.5 M EDTA (pH 7.6) stock, and a 1:20 dilution of Streck Cell-Free DNA urine preserve. Urine from TB patients in Uganda was preserved in 10 mM Tris-HCl–10 mM EDTA (pH 8.5) and Streck Cell-Free DNA urine preserve, frozen at –80°C, and shipped to Stanford University for testing.

### Processing delay.

One set of blood collection tubes and urine specimens was immediately processed. The second and third sets were processed after room temperature incubation periods of 6 and 24 h, respectively. All remaining procedures were identical for the three sets.

### Processing method.

For single-spin plasma separation, blood collection tubes were centrifuged at 500 × *g* for 10 min at room temperature, and the plasma was transferred to a new tube. For double-spin plasma separation, 1.5 ml of plasma was additionally centrifuged at 16,000 × *g* for 10 min at room temperature, and the supernatant was transferred to a new tube. For urine processing, whole urine was centrifuged at 500 × *g* for 10 min, and the supernatant was transferred to a new tube.

### Fresh versus thawed.

Plasma obtained through single-spin plasma separation and whole urine from the spiking experiments were stored at –80°C for 1 and/or 24 weeks. Samples were thawed at room temperature and extracted for comparison to fresh samples.

### Sample volume.

Blood collected in K_2_EDTA tubes and EDTA-urine from five healthy donors were spiked with M. tuberculosis cfDNA at the highest detectable dilution (see “Pathogen cfDNA preparation,” above) and at a 10-fold higher concentration. Blood was processed using single-spin centrifugation. One and 4 ml of fresh EDTA-plasma and whole urine were extracted using the Maxwell RSC system. The Maxwell RSC ccfDNA plasma kit and a custom Maxwell RSC large-volume ccfDNA kit available commercially were used to extract 1 and 4 ml, respectively. Sample volume was also investigated in TB patients using EDTA-plasma from patients in Brazil and Tris-EDTA-urine from patients in Uganda. After thawing samples, blood samples were extracted with QIAamp circulating nucleic acid kit (Qiagen), and urine samples were extracted with the Maxwell RSC system.

### cfDNA measurement.

Except for assessments of sample volume, 1 ml each of plasma and urine were extracted at each time point using the Maxwell RSC ccfDNA plasma kit. The real-time PCR conditions described above were used to determine *C_T_* values for M. tuberculosis, S. enterica, A. fumigatus, and EBV in spiking experiments and M. tuberculosis in clinical samples. Each PCR was performed in singlicate. The median *C_T_* values, a measure of amplifiable cfDNA, were compared between different conditions. Except for fresh versus thawed experiments, all cfDNA extracts from the same individual’s plasma and urine were tested in the same PCR run.

### Statistical analysis.

A nonparametric test, the Wilcoxon signed-rank test of medians, was used to compare differences between paired results. The EDTA group was used as the comparator for all analyses. All statistical tests were computed for a two-sided type I error rate of 5%. Statistical analyses were performed using the Prism software (GraphPad, San Diego, CA).

## RESULTS

### Blood collection tube and urine preservative.

**FIG 2:**
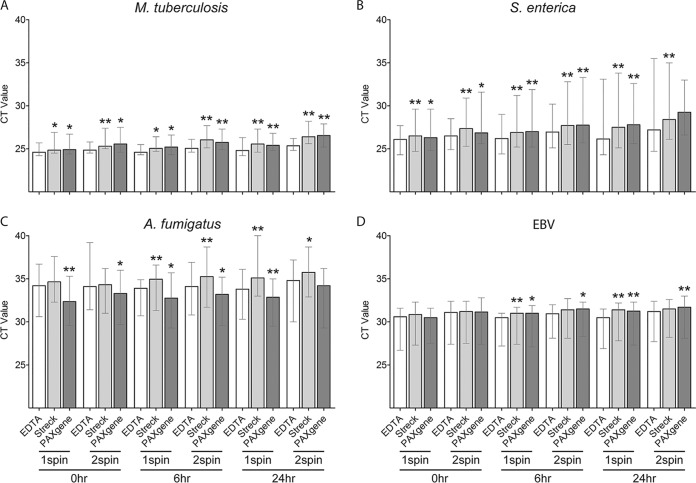
Comparison of blood collection tubes for recovery of pathogen cfDNA in plasma using contrived samples. (A to D) Blood was collected from 10 healthy donors in a K_2_EDTA (EDTA) tube, Streck Cell-Free DNA BCT, and a PAXgene blood ccfDNA tube and spiked with short fragments of DNA from M. tuberculosis (A), S. enterica (B), A. fumigatus (C), and EBV (D). Blood collection tubes were processed after 0-, 6-, and 24-h delays at room temperature, and plasma was obtained using one-spin (1spin) and double-spin (2spin) separation. PCR was performed on cfDNA extracts. Bars show median *C_T_*, and whiskers show the *C_T_* range. For each condition (processing time and plasma separation method), Streck and PAXgene tubes were compared to EDTA tubes. *, *P* < 0.05; **, *P* < 0.01.

A comparison of the standard K_2_EDTA tube to two cfDNA blood collection tubes spiked with pathogen cfDNA and processed identically for plasma separation and extraction showed a significantly lower median *C_T_* value with K_2_EDTA tubes for M. tuberculosis and S. enterica than with Streck and PAXgene tubes (Fig. 2A and B and Table S2). The median A. fumigatus *C_T_* was significantly lower for K_2_EDTA tubes than with Streck tubes but only after 6- and 24-h processing delays (Fig. 2C). The median A. fumigatus *C_T_* was significantly lower for PAXgene tubes than for K_2_EDTA tubes except for the 24-h processing delay with double-spin plasma separation (Fig. 2C). The median EBV *C_T_* was lower for K_2_EDTA tubes than for Streck and PAXgene tubes, but the difference was significant only after 6- and 24-h processing delays for single- and/or double-spin plasma separation (Fig. 2D).

**FIG 3:**
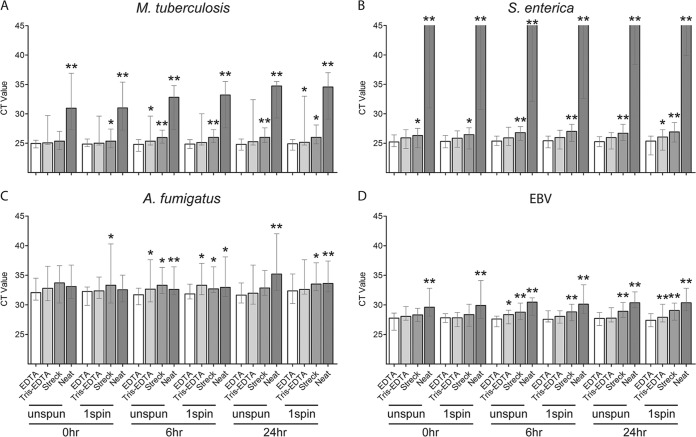
Comparison of urine preservatives for recovery of pathogen cfDNA using contrived samples. (A to D) Urine was collected from 10 healthy donors and preserved with 25 mM EDTA, 10 mM Tris-EDTA, or Streck Cell-Free DNA urine preserve, or left unpreserved (neat urine) and spiked with short fragments of DNA from M. tuberculosis (A), S. enterica (B), A. fumigatus (C), and EBV (D). Urine samples were processed after 0-, 6-, and 24-h delays at room temperature. Whole urine (unspun) and urine supernatant (1spin) were included. PCR was performed on cfDNA extracts. Bars show median *C_T_*, and whiskers show the *C_T_* range. In the absence of amplification for S. enterica in neat urine, a *C_T_* of 45 was assigned. For each condition (processing time and urine processing method), Tris-EDTA, Streck, and neat were compared to EDTA. *, *P* < 0.05; **, *P* < 0.01.

A comparison of three urine preservatives using urine specimens from healthy donors spiked with pathogen cfDNA showed significantly lower median pathogen *C_T_* values for 25 mM EDTA than with Streck urine preservative for all four pathogens at all time points for whole urine (unspun) and/or urine supernatant (one spin), with the exception of EBV, which was significantly lower only after 6- and 24-h processing delays (Fig. 3 and Table S3). In many instances, 25 mM EDTA yielded a lower median *C_T_* than did 10 mM Tris-EDTA, but this difference reached statistical significance only after 6- and/or 24-h processing delays for whole urine and/or urine supernatant (Fig. 3). Unpreserved (neat) urine consistently yielded a significantly higher median *C_T_* than did 25 mM EDTA for all four pathogens at all time points, with the exception of A. fumigatus, which was significant only after 6- and 24-h processing delays (Fig. 3 and Table S3).

**FIG 4:**
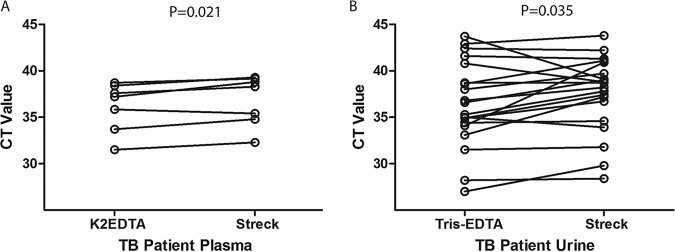
Comparison of blood collection tubes and urine preservatives for detection of M. tuberculosis cfDNA in plasma and urine from patients with tuberculosis. (A and B) IS*6110* PCR *C_T_* values are plotted for plasma samples collected concurrently in K_2_EDTA and Streck Cell-Free DNA BCT (*n* = 7) (A), and urine samples concurrently preserved in Tris-EDTA and Streck Cell-Free DNA urine preserve (*n* = 20) (B). Results are shown for patients with detectable M. tuberculosis cfDNA with both collection tubes and preservatives.

In patients with pulmonary TB and detectable M. tuberculosis cfDNA in plasma, blood samples concurrently collected in K_2_EDTA and Streck tubes and processed immediately showed significantly lower median M. tuberculosis *C_T_* values with K_2_EDTA-plasma than with Streck-plasma (*P* = 0.021) (Fig. 4A and Table S4). In patients with pulmonary TB and detectable M. tuberculosis cfDNA in urine, urine samples concurrently preserved with 10 mM Tris-EDTA and Streck urine preserve showed significantly lower median M. tuberculosis *C_T_* with 10 mM Tris-EDTA than with Streck urine preservative (*P* = 0.035) (Fig. 4B and Table S4).

### Processing delay.

**FIG 5:**
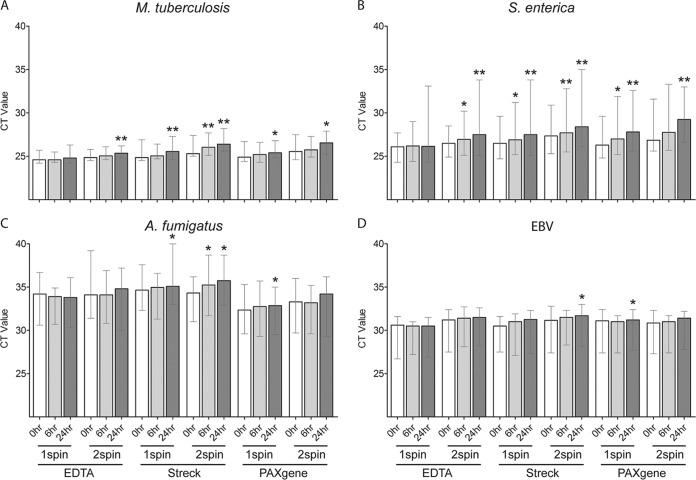
Impact of processing delay on recovery of pathogen cfDNA in plasma using contrived samples. (A to D) Blood was collected from 10 healthy donors in a K_2_EDTA tube, Streck Cell-Free DNA BCT, and PAXgene blood ccfDNA tube and spiked with short fragments of DNA from M. tuberculosis (A), S. enterica (B), A. fumigatus (C), and EBV (D). Blood collection tubes were processed after 0-, 6-, and 24-h delays at room temperature, and plasma was obtained using one-spin (1spin) and double-spin (2spin) separation. PCR was performed on cfDNA extracts. The bars show the median *C_T_*, and whiskers show the *C_T_* range. For each condition (blood collection tube and plasma separation method), 6- and 24-h processing delays were compared to 0 h. *, *P* < 0.05; **, *P* < 0.01.

Compared to immediate processing (i.e., 0-h delay) of blood collection tubes for plasma separation, a delay of 6 and/or 24 h at room temperature increased the median pathogen *C_T_* values of all four pathogens spiked in Streck and PAXgene tubes after single- and/or double-spin plasma separation, although the difference was only consistently significant for M. tuberculosis and S. enterica (Fig. 5 and Table S5). Processing delays of 6 and 24 h with single-spin plasma separation did not significantly change the median *C_T_* values of any of the four spiked pathogens in a K_2_EDTA tube (Fig. 5 and Table S5).

**FIG 6:**
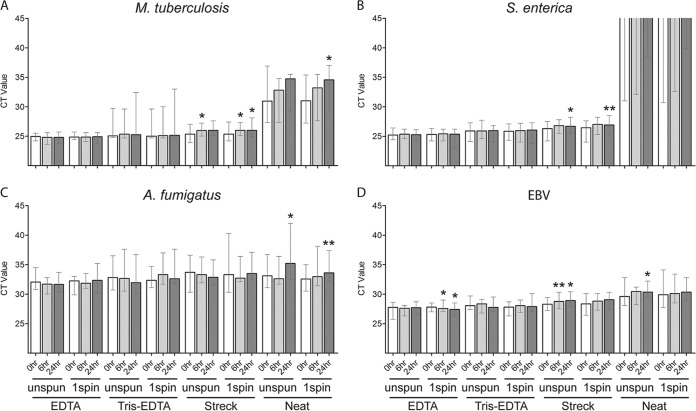
Impact of processing delay on recovery of pathogen cfDNA in urine using contrived samples. (A to D) Urine was collected from 10 healthy donors and preserved with 25 mM EDTA, 10 mM Tris-EDTA, Streck Cell-Free DNA urine preserve, or left unpreserved (neat urine) and spiked with short fragments of DNA from M. tuberculosis (A), S. enterica (B), A. fumigatus (C), and EBV (D). Urine samples were processed after 0-, 6-, and 24-h delays at room temperature. Whole urine (unspun) and urine supernatant (1spin) were evaluated. PCR was performed on cfDNA extracts. The bars show median *C_T_* values, and whiskers show the *C_T_* range. In the absence of amplification for S. enterica in neat urine, a *C_T_* of 45 was assigned. For each condition (urine preservative and urine processing method) 6- and 24-h processing delays were compared to 0 h. *, *P* < 0.05; **, *P* < 0.01.

For urine samples, processing delays of 6 and 24 h did not increase the median *C_T_* values of any of the four pathogens in urine preserved with 25 mM EDTA and 10 mM Tris-EDTA (Fig. 6 and Table S6). Preservation of urine with Streck preservative resulted in a significant increase in median *C_T_* values of M. tuberculosis, S. enterica, and EBV after 6- and/or 24-h delays in unspun urine and/or urine supernatant (1 spin). With unpreserved (neat) urine, the median *C_T_* increased significantly for M. tuberculosis, A. fumigatus, and EBV, which was statistically significant after a 24-h delay in whole urine and/or supernatant. In neat urine spiked with S. enterica, most samples had undetectable PCR amplification at all time points.

### Processing method.

**FIG 7:**
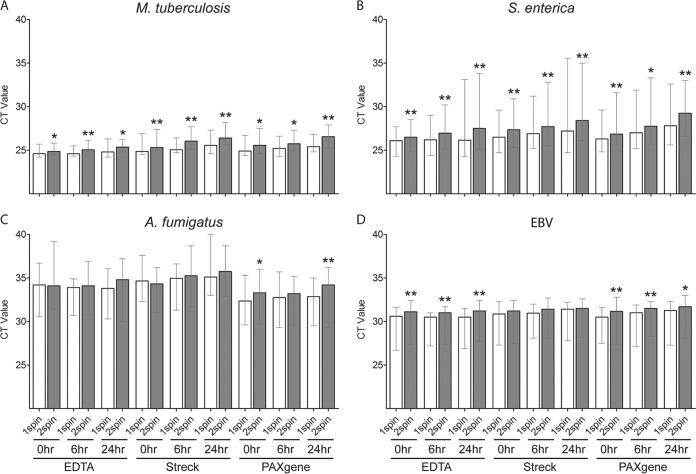
Comparison of single-spin to double-spin plasma separation for recovery of pathogen cfDNA using contrived samples. (A to D) Blood was collected from 10 healthy donors in a K_2_EDTA tube, Streck Cell-Free DNA BCT, and PAXgene Blood ccfDNA tube and spiked with short fragments of DNA from M. tuberculosis (A), S. enterica (B), A. fumigatus (C), and EBV (D). Blood collection tubes were processed after 0-, 6-, and 24-h delays at room temperature, and plasma was obtained using one-spin (1spin) and double-spin (2spin) separation. PCR was performed on cfDNA extracts. Bars show median *C_T_* values, and whiskers show the *C_T_* range. For each condition (blood collection tube and processing delay), double spin was compared to one spin. *, *P* < 0.05; **, *P* < 0.01.

A comparison of single-spin and double-spin plasma separation methods showed lower median *C_T_* values of all four pathogens with single-spin separation than with double-spin separation, except for A. fumigatus in K_2_EDTA and Streck tubes after a 0-h processing delay (Fig. 7 and Table S7). The difference was statistically significant for M. tuberculosis, S. enterica, and EBV at all three time points (0-, 6-, and 24-h processing delays) and with all three blood collection tubes, with the exception of EBV, which was only significant with K_2_EDTA and PAXgene tubes. For A. fumigatus, the difference was statistically significant only after 0- and 24-h processing delays with the PAXgene tube.

A comparison of whole urine (unspun) to urine supernatant (one spin) showed no consistent difference in median *C_T_* values of all four pathogens at all three time points (0-, 6-, and 24-h processing delays) and with all three urine preservatives (Fig. S1 and Table S8).

### Fresh versus thawed.

A comparison of fresh and thawed plasma after 1 and 24 weeks of storage at –80°C showed no significant difference in median *C_T_* values of all four pathogens after immediate processing with all three blood collection tubes, with the exception of S. enterica in a PAXgene tube (Fig. S2 and Table S9).

A comparison of fresh and thawed urine after 24 weeks of storage at –80°C showed no difference in median *C_T_* values of all four pathogens after immediate processing with all three urine preservatives (Fig. S3 and Table S10).

### Sample volume.

**FIG 8:**
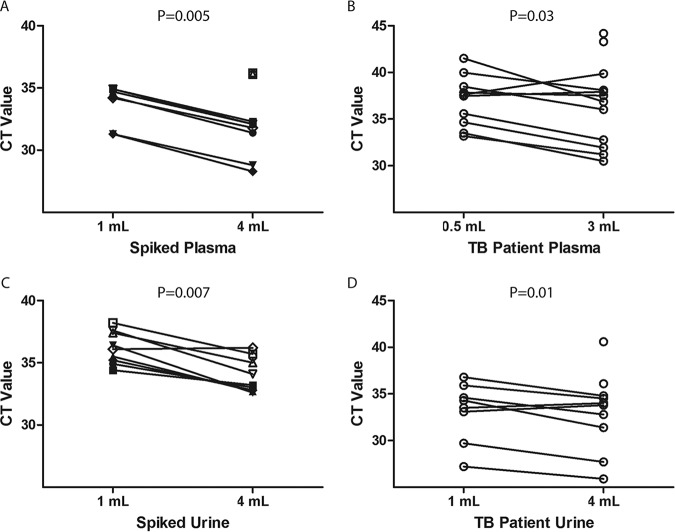
Comparison of small- and large-volume plasma and urine for detection of M. tuberculosis cfDNA using contrived samples and tuberculosis patient samples. (A and C) Spiked plasma (A) and urine (C) samples from five healthy donors (unique symbols). Blood samples were collected in a K_2_EDTA tube, and urine samples were preserved with 25 mM EDTA. Blood and urine samples were spiked with short fragments of DNA from M. tuberculosis at the highest detectable dilution (open symbols) and at 10-fold higher concentration (filled symbols). (B and D) K_2_EDTA plasma (*n* = 12) (B) and Tris-EDTA urine (*n* = 10) (D) samples from tuberculosis patients with detectable cfDNA. IS*6110* PCR was performed on cfDNA extracts, and *C_T_* values were plotted. Unpaired symbols indicate that a positive result was only observed with a larger volume.

A comparison of small and large volumes of plasma and urine for the detection of M. tuberculosis cfDNA using contrived samples (1 ml versus 4 ml) and clinical samples from newly diagnosed TB patients (plasma, 0.5 ml versus 3.0 ml; urine, 1 ml versus 4 ml) showed a significantly lower median *C_T_* with larger volumes for both plasma and urine (Fig. 8 and Table S11). In a fraction of contrived and clinical samples, amplification was only detected with the larger sample volume (Fig. 8).

## DISCUSSION

The detection of pathogen cfDNA in plasma and urine potentially affords an attractive novel noninvasive approach to diagnosing invasive infections. However, little is known about the impact of preanalytical factors, such as type of blood collection tube or urine preservative, processing delay, processing method, sample volume, and freezing and thawing, on the detection of pathogen cfDNA (23). We showed that most of the preanalytical factors deemed important for fetal and tumor cfDNA (24–26, 28) do not seem to apply to pathogen cfDNA. These results increase the potential for low-cost pathogen cfDNA assays to be developed for infectious disease diagnostics.

Using blood and urine samples spiked with cfDNA from four different pathogens representative of bacteria, fungi, and DNA viruses, we showed the standard K_2_EDTA blood collection tube, which is inexpensive and widely available, yields an amount of detectable pathogen cfDNA higher than or equivalent to that of Streck and PAXgene tubes, except for A. fumigatus cfDNA in PAXgene tubes. Importantly, the addition of preservative to urine was critical to preventing the degradation of pathogen cfDNA, and preservation with 25 mM EDTA was superior to Streck urine preservative. We were also able to confirm the superiority of K_2_EDTA and 25 mM EDTA over Streck tubes using plasma and urine, respectively, from patients with active TB. Similar to tumor cfDNA in plasma (25), we showed that pathogen cfDNA in blood collected in K_2_EDTA and urine preserved with 25 mM EDTA is stable for at least 24 h at room temperature. Unlike tumor cfDNA, for which double-spin plasma separation helps prevent tumor cfDNA dilution after delayed processing (26), a single low-speed centrifugation was sufficient to maximize the yield of spiked pathogen cfDNA up to 24 h after sample collection. With urine samples, we showed that separation of cellular fraction from whole urine with a centrifugation step had no impact on the yield of pathogen cfDNA up to 24 h after urine collection. In agreement with tumor cfDNA, extraction of a higher volume of plasma and urine yielded a higher abundance of M. tuberculosis cfDNA in contrived samples and samples collected from TB patients. Last, freezing and thawing of plasma and urine samples after storage at –80°C up to 24 weeks did not have any impact on the abundance of pathogen cfDNA in spiked samples.

Our findings bear implications for the application of pathogen cfDNA in the clinical laboratory for the diagnosis of invasive infections, particularly in resource-limited settings. The findings that the K_2_EDTA blood collection tube combined with single-spin low-speed plasma separation and 24-h processing delay yields the maximum amount of amplifiable cfDNA for 3 out of 4 pathogens we evaluated implies that optimized preanalytical steps can be feasibly and inexpensively implemented in the routine microbiology laboratory workflow, both in resource-rich and resource-limited settings. Unlike Streck and PAXgene cfDNA blood collection tubes, which cost about $10 each, K_2_EDTA tubes cost less than $0.50. Similarly, the findings that 25 mM EDTA preserves pathogen cfDNA in whole urine without the need to remove cellular debris with centrifugation and up to a 24-h processing delay implies that optimized preanalytical steps can be inexpensively operationalized for pathogen cfDNA testing in urine.

Although the findings of this study are promising, a better understanding of the underlying biochemical basis of preanalytical factors impacting cfDNA recovery may lead to further optimization and improvement of cfDNA testing. The finding that the K_2_EDTA blood collection tube was superior to the Streck and PAXgene tubes for the recovery of cfDNA from M. tuberculosis, S. enterica, and EBV after delayed processing may be explained by EDTA (1.8 mg/ml) in K_2_EDTA, which protects pathogen cfDNA from endogenous DNase activity in blood (31). Whether increasing the EDTA concentration by as much as 10-fold, as suggested by Barra and colleagues to fully inhibit endogenous DNase activity (31), can further increase the yield of pathogen cfDNA remains to be shown. Another interesting finding was that more A. fumigatus cfDNA was recovered from PAXgene tubes than from K_2_EDTA tubes. Whether this can be reproduced in patients with invasive fungal disease and the molecular basis of this result have important implications for the sensitivity of cfDNA assays used to diagnose invasive fungal disease (13, 15). We also observed a higher pathogen cfDNA yield after single-spin plasma separation than with double-spin separation. The molecular basis underlying this finding is unclear, but understanding it may facilitate designing a novel tube with higher cfDNA yield. Last, the difference in urine cfDNA stability between pathogens is intriguing. While M. tuberculosis and S. enterica cfDNA were rapidly degraded in unpreserved urine, A. fumigatus and EBV cfDNA was less prone to degradation. Whether this is due to differences in DNA packaging between prokaryotic and eukaryotic organisms remains to be determined.

This study has several limitations. First, although the variables investigated using TB patient samples in this study correlated very well with findings from contrived samples, not all findings from contrived samples could be confirmed. As such, further studies are needed to validate our findings with clinical samples. Second, although many of the findings with spiked samples were statistically significant, there was an overlap between groups in most cases. Thus, the clinical significance of our findings needs to be further investigated in clinical studies. Third, we did not include a serum collection tube to compare serum to plasma for the recovery of pathogen cfDNA. This was because serum has been shown to have 15-fold higher endogenous nuclease activity and a smaller fraction and quantity of tumor and fetal cfDNA, respectively, than with plasma (25, 31). Importantly, the sensitivity of *Aspergillus* PCR was shown to be higher in plasma than in serum (94.7% versus 68.4%, respectively) (13). We also did not investigate heparin as the anticoagulant for plasma because older studies had shown that it inhibits PCR (32–34). Fourth, we did not investigate processing delays beyond 24 h. However, 24 h is a sufficient time period to collect and transport samples to the laboratory for processing in most institutions. Last, we did not compare extraction methods for pathogen cfDNA. This was the topic of investigation of a recent study that evaluated commercial methods available for the extraction of plasma cfDNA (35). A key finding from this study was that commercial methods are biased toward longer cfDNA. Further studies are needed to investigate and optimize the extraction of pathogen cfDNA from plasma and urine.

In summary, we evaluated preanalytical factors impacting the recovery of pathogen cfDNA from blood and urine and found that large-volume single-spin K_2_EDTA-plasma and EDTA-whole urine with up to a 24-h processing delay represent good choices for pathogen cfDNA. Future studies can focus on measuring the performance of pathogen cfDNA assays using optimized preanalytical factors described here. It is likely that more efficient pathogen cfDNA extraction methods and sensitive cfDNA NAATs, ideally, sample-to-answer tests, are needed to complement the preanalytical optimization steps described in this study in order to move noninvasive diagnosis of invasive infections into routine practice.

## Supplementary Material

Supplemental file 1
